# Marine-Derived Biomolecules

**DOI:** 10.3390/biom11010012

**Published:** 2020-12-25

**Authors:** Diaa T. A. Youssef

**Affiliations:** 1 Department of Natural Products, Faculty of Pharmacy, King Abdulaziz University, Jeddah 21589, Saudi Arabia; dyoussef@kau.edu.sa; 2 Department of Pharmacognosy, Faculty of Pharmacy, Suez Canal University, Ismailia 41522, Egypt

The world’s oceans have been shown to be rich habitats with great biodiversity and chemical entities with proven bioactivities related to cancer, inflammation, epilepsy, the immunomodulatory system, microbial and parasitic infections, and many others. Currently, there are eight approved drugs of marine origin and more than 22 other chemical entities in different clinical phases. Marine invertebrates and micro-organisms represent the major sources for these compounds. The advantages of studying organisms from the marine environment lie primarily in the breadth of marine biodiversity and the consequent variety of new chemical structures found among marine natural products. Attesting to the tremendous diversity of marine life is the fact that, of the 33 animal phyla, 32 are found in the sea, while only 12 occur on land. The larger genetic pool found in the marine environment has resulted in the synthesis of a wide variety of chemicals that can be exploited in a systematic screening program. Many of the primitive phyla that have evolved over the longest times in the sea appear to have done so using survival mechanisms based on chemical synthesis. The compounds that are responsible for the successful survival of marine organisms possess significant biological activities that often interfere with the essential growth or biosynthetic mechanisms of competing organisms. These are precisely the types of chemicals that might be expected to be active in cancer-related bioassays. Over many millions of years of evolution, marine animals have evolved molecules with high binding affinities toward intracellular targets. The opportunity to apply these “evolutionarily significant molecules” within a mechanism-based drug discovery program is thus a rational approach to targeted drug discovery.

The scope of this Special Issue is to provide a broad and updated overview on marine-derived biomolecules and their diverse bioactivities as potential drug leads. The collection includes seven original research papers from prominent researchers in the field and provides the readers of the journal with recent results in the area of marine pharmacology and biomedicine. Therefore, this Special Issue promotes our understanding of marine-derived biomolecules and the role that they could play as a future resource for drug discovery. 

The study by Shaala and Youssef [1] explored the cytotoxic principles of the Verongid sponge *Aplysinella* species. They identified two new psammaplysin derivatives, psammaplysin Z and 19-hydroxypsammaplysin Z, along with the previously reported psammaplysins, A and E. They showed that psammaplysins A and E exhibit cytotoxic activity against MBA-MB-231 and HeLa cell lines with IC_50_ values down to 0.29 µM, while psammaplysin Z and 19-hydroxypsammaplysin Z were less cytotoxic, suggesting the importance of the terminal amine (in psammaplysin A) and 2-(methylene)cyclopent-4-ene-1,3-dione moieties (in psammalysin E) for potent cytotoxic activities.

The article by Rey-Campos et al. [2] studied hemocytes’ transcriptomic responses induced by myticin C treatment. The study concluded that mycitin C promotes changes in the expression profile and mobility behavior of hemocytes. They claimed that these changes are of consequence to the great number of genes directly related to the actin cytoskeleton, which are modulated by the peptide. Further, myticin C seems to accelerate all processes of regeneration in tissue injury. This would support the existing theory that myticin C is a cytokine-like protein exclusive to mussels.

In the article by Ahn et al. [3], the authors purified butyrolactone I from the marine fungus *Aspergillus terreus* and found that the compound targets CDKs and PPARγ. They claimed that butyrolactone I possesses an additive or synergistic therapeutic potential in diseases with multifactorial etiologies. In addition, the polypharmacophore of butyrolactone I and the crystal structure complexed with PPARγ LBD offer better opportunities to design a novel PPARγ partial agonist expecting therapeutic synergism. 

Llorach-Pares et al. [4] showed, by computational and experimental studies, that meridianins and lignarenone B can inhibit the activity of GSK3β, likely through an adenosine triphosphate (ATP) competitive and noncompetitive allosteric mechanism. Furthermore, they claim that these compounds can increase neurite outgrowth in primary cortical neurons without neurotoxicity. Once the compounds entered the cells, they showed a good inhibitory profile, and a good permeability toward the cellular membrane, and so should be able to penetrate the brain. 

Zdarta et al. [5] studied the effect of 3D chitin scaffolds from the marine sponge *Aplysina archeri* as a support for laccase immobilization and their application in the removal of pharmaceuticals. They report on the use of this chitin for adsorption and immobilization of laccase from *Trametes versicolor*. Moreover, they showed that the thermal and storage stabilities of the immobilized enzyme were significantly improved as compared to the free enzyme, indicating the protective effect of the chitinous support on the biomolecules.

The work by Amaral et al. [6] focused on the preparation and characterization of mucoadhesive insulin-loaded polymeric nanoparticles. The results showed a suitable mean size for oral administration (<600 nm by dynamic laser scattering), spherical shape, encapsulation efficiency (59.8%), and high recovery yield (80.6%). Additionally, they showed that insulin-loaded nanoparticles are effective in reducing diabetic rats’ glycemia. Finally, they claim that the coating of insulin-loaded nanoparticles with chitosan represents a potentially safe and promising approach to protect insulin and enhance peroral delivery.

An article by Patel et al. [7] focused on the investigation of the unexplored mucus extract of *Puntius sophore* (*P. sophore*) for its antagonistic potential against common pathogens. They showed that the mucus extract of *P. sophore* is effective against all tested strains. Additionally, *P. sophore* mucus extract was found to inhibit biofilm formation by affecting the viability and integrity of bacterial cells within biofilms, as well as by hampering the production of Extracellular Polysaccharide (EPS). *P. sophore* mucus extract showed synergetic effect with gentamicin against several pathogens. 

This Special Issue describes important findings related to the general and diverse bioactivities of marine-derived compounds, as well as their potential use and application in different areas related to human diseases. The issue also highpoints the most recent advancements in the knowledge and the pharmacological investigations of the compounds. This may be helpful in assessing prognostic or predictive indicators, as well as developing new therapies and new insights aimed at improving lifestyle. 
